# Formation and dynamics of a solar eruptive flux tube

**DOI:** 10.1038/s41467-017-02616-8

**Published:** 2018-01-12

**Authors:** Satoshi Inoue, Kanya Kusano, Jörg Büchner, Jan Skála

**Affiliations:** 1 Max-Planck Institute for Solar System Research, Justus-von-Liebig-Weg 3, 37077 Göttingen, Germany; 2 Institute for Space-Earth Environmental Research, Nagoya University, Furo-cho, Chikusa-ku, Nagoya 464-8601 Japan

## Abstract

Solar eruptions are well-known drivers of extreme space weather, which can greatly disturb the Earth’s magnetosphere and ionosphere. The triggering process and initial dynamics of these eruptions are still an area of intense study. Here we perform a magnetohydrodynamic simulation taking into account the observed photospheric magnetic field to reveal the dynamics of a solar eruption in a real magnetic environment. In our simulation, we confirmed that tether-cutting reconnection occurring locally above the polarity inversion line creates a twisted flux tube, which is lifted into a toroidal unstable area where it loses equilibrium, destroying the force-free state, and driving the eruption. Consequently, a more highly twisted flux tube is built up during this initial phase, which can be further accelerated even when it returns to a stable area. We suggest that a nonlinear positive feedback process between the flux tube evolution and reconnection is the key to ensure this extra acceleration.

## Abstract (web summary)

Solar eruptions are large explosions occurring in the solar atmosphere. Here, the authors perform magnetohydrodynamic simulations to unveil the dynamics of a solar eruption, and find that these are dominated by nonlinear processes involving flux tube evolution and reconnection.

## Introduction

Solar eruptions are the largest explosions observed in the solar atmosphere and the driver source is widely believed to be magnetic flux tubes^1^, which are composed of helical twisted field lines. Due to the electric current inside a magnetic flux tube, a Lorentz force can be exerted on the flux tube and induce current driven instability into the system^2,3^. In order to reveal the formation and eruptive dynamics of the solar flux tubes, a lot of effort have been made to construct models of two- and three-dimensional (3D) flux tubes before and also during eruptions^4–7^ because, unfortunately, direct observation of the coronal magnetic fields is not yet possible. In addition, the latest telescopes onboard satellites and ground-based observatories clearly show much evidence of flux tubes on the solar surface as well as those eruptions^8,9^. These observational and theoretical studies have deepened our understanding of this phenomena.

Recently, the torus instability (TI)^10^ is a strong candidate for explaining the initiation of the flux tube and slow rise to fast acceleration of solar eruptions^11–13^. Torus instability can be triggered when the upward hoop force from electric current flowing inside of the flux tube dominates the downward strapping force derived from the external magnetic fields. The decay index (*n*: see method), which is a measure of the extrenal horizontal field decreases, is then important to determine the dynamics^14^. The stability analysis suggests that the TI can grow when *n* ≥ 1.5^10^ and recent numerical studies point out that the critical decay index may be in the range of *n* = 1.3–1.5^15^. However, several studies argued that the decay index does not always control the dynamics. For instance, laboratory experiments^16^ showed that the flux tube evolution is halted even though *n* ≥ 1.5 condition is satisfied and launch away from bottom. Because a dynamic tension force working in nonlinear regime is important to produce the downward force on the flux tube and strongly brake the ascension even in the region satisfying *n* ≥ 1.5^17^. Furthermore, recent observations^18,19^ confirmed several eruptions occurred even though the decay index takes the saddle-like profile where the flux tube starts launching in an area (*n* ≥ 1.5), but later returns into TI-stable area (*n* ≤ 1.5). Nevertheless, the flux tube shows continuous ascension without halt and eventually can reach another area satisfying the *n* ≥ 1.5 condition. Although the several eruptions in saddle-like decay index profiles are reported, the physical mechanism why the flux tube can pass through TI-stable regions and be accelerated is not yet clear.

In order to clarify the dynamics of solar eruptive flux tube in that situation, here we perform the MHD simulation using the photospheric magnetic field from which the initial condition is reconstructed in the nonlinear-force-free field (NLFFF) approximation^20,21^. This was done to shorten the distance between theoretical models and observations, and construct a more realistic magnetic environment. We observed a solar eruption on 13 February 2011 occurred in solar active region 11158^22^, producing an M6.6 class flare and associated the coronal mass ejection (CME). A saddle-like profile of the decay index is also observed in the height of the decay index in which the flux tube undergoes the dynamics and go through TI-stable area. Some kind of nonlinear process would be important^16,17^ to exclude the flux tube from TI-stable area. Our simulation first shows that the highly twisted flux tube is buildup during the eruption in earlier phase. The magnetic reconnection is then a key to generate that twisted flux tube. Eventually, we conclude that the nonlinear process of the flux tube evolution, coupling with the reconnection is significant for driving the eruption, in particular, we found the dramatic acceleration of the flux tube even in TI-table area. We present detailed analysis of this eruptive flux tube in following section.

## Results

### Initial state prior to the eruption

**Fig. 1:**
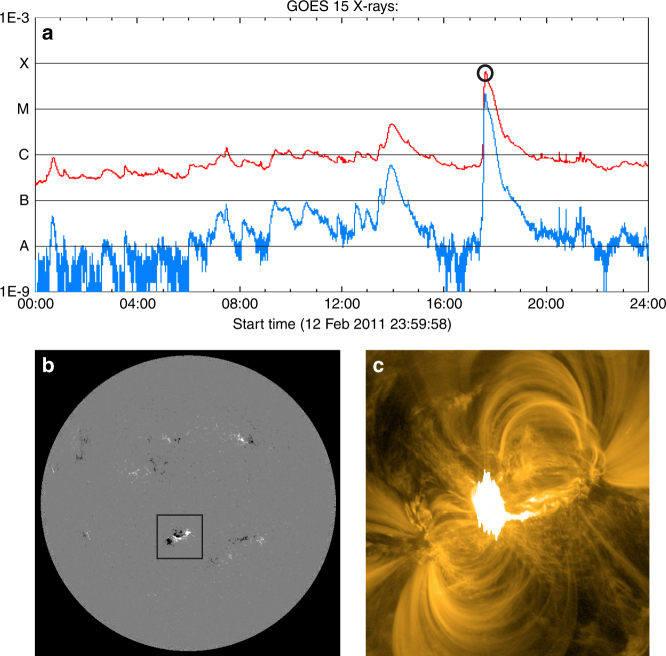
M6.6 class solar flare observed on 2011 February 13. **a** Time profile of the X-ray flux measured by the GOES 12 satellite on February 13. The M6.6 class flare marked by the open circle started at 17:28 UT and the peak was observed at 17:30 UT. The solar X-ray outputs in the 1–8 and 0.5–4.0 Å passband are plotted, respectively. **b** The line-of-sight magnetic field of whole sun observed at 17:48 UT taken from SDO/HMI. The cluster of the magnetic flux surrounded by the black square corresponds to the AR11158 producing the flare. **c**, EUV 171 Å image of the M6.6-class solar flare taken by SDO/AIA observed at 17:45 UT

**Fig. 2:**
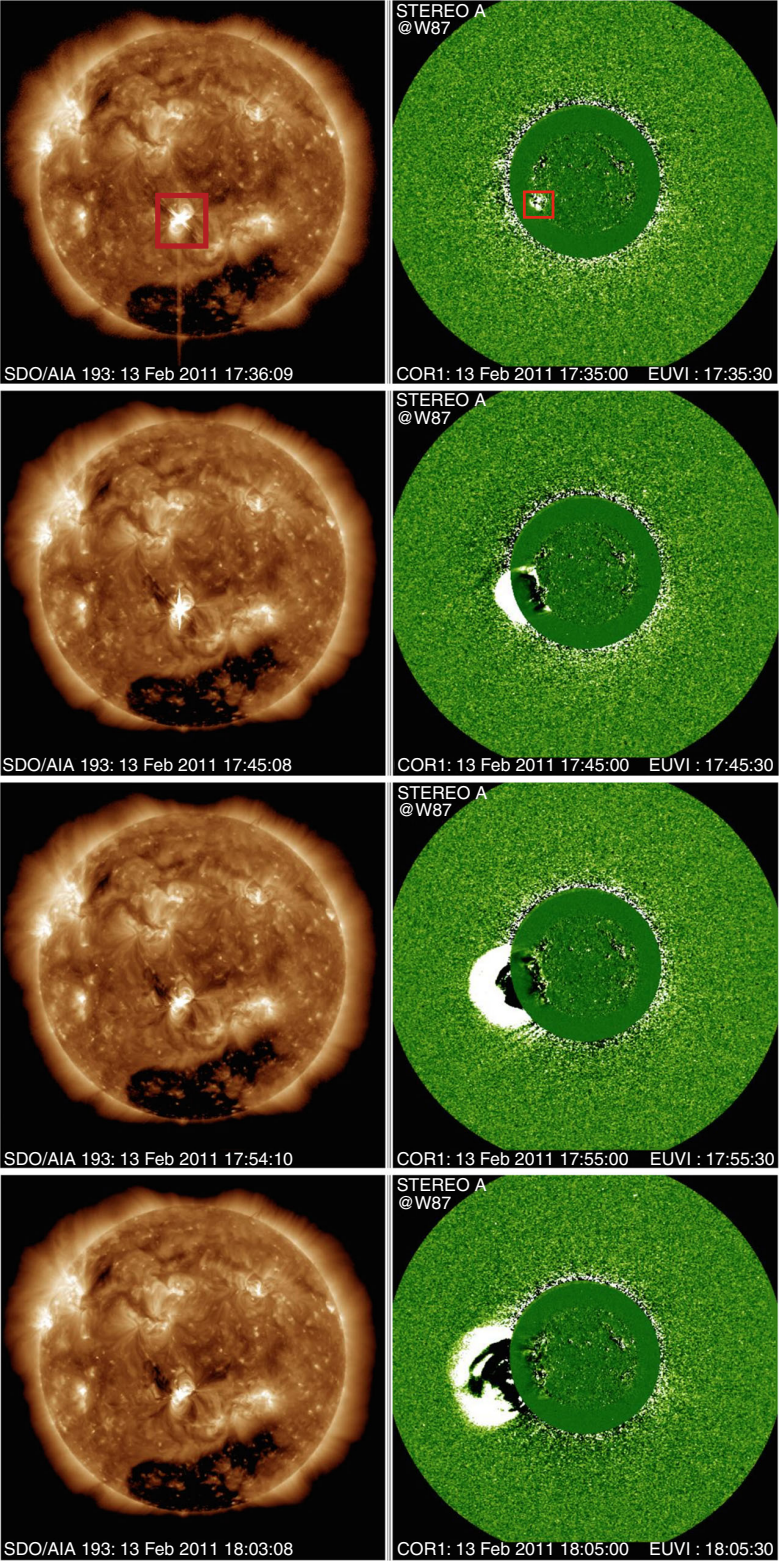
CME observation associated with the M6.6-class solar flare on 13 February 2011. The left panels show the temporal evolution of the whole sun observed with EUV 193 Å taken from SDO/AIA. The M6.6 class flare was observed in the red square. CME is clearly observed by STEREO-A, associated with the M6.6 class flare. These figures are courtesy from Dr. S. Yashiro (http://cdaw.gsfc.nasa.gov/CMElist/)

**Fig. 3:**
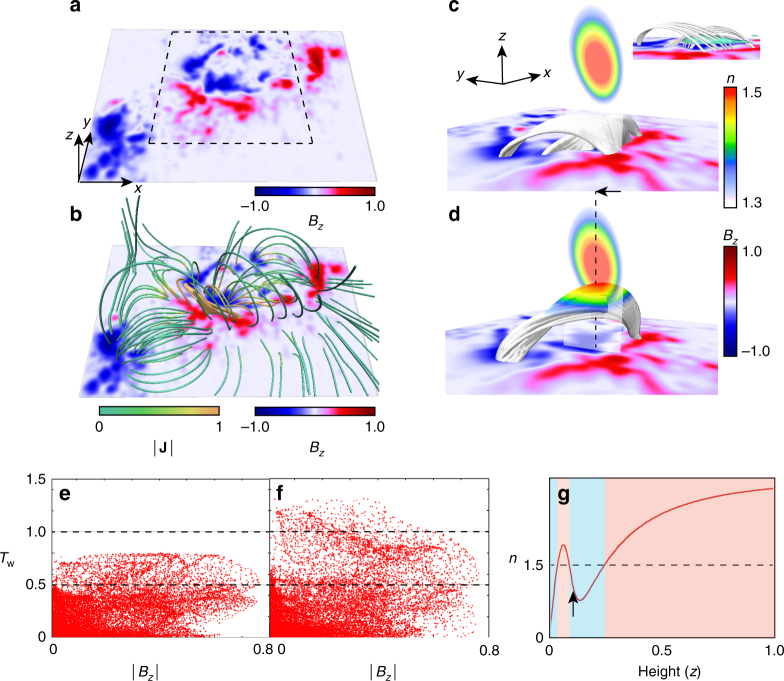
Stability of the magnetic fields before and after the tether-cutting reconnection. **a** Photospheric magnetic field (*B*_*z*_) of AR11158 taken by SDO/HMI observed at 16:00 UT on February 13, corresponding to 90 min approximately before the M6.6-class flare. The NLFFF and twist are calculated only from the region surrounded by the dashed-square in the range of 0.38 L* ≥ *x* ≥ 0.65 L* and 0.38 L* ≥ *y* ≥ 0.65 L*, where the outside of the boundary is fixed by the potential field. **b** Three-dimensional field lines of the NLFFF, which are represented with the current density |**J**|. **c** The field lines of the NLFFF, which focuses on the twisted lines enhanced with |**J**| in **b**. The color of the field lines and vertical cross section corresponds to the decay index. The small inset shows the side view with green isosurface of |**J**| = 30. **d** The field lines after the tether-cutting reconnection. **e**, **f** Twist (*T*_w_) distribution of the NLFFF and a state after the tether-cutting reconnection, depending on |*B*_*z*_| measured at each footpoint of the field lines. **g** The decay index depending on the height measured along the vertical dashed line in **d**, where the horizontal dashed line marks *n* = 1.5. Red and blue regions indicate the unstable and stable those to the torus instability, i.e., a flux tube loses the equilibrium in a region marked in red. The arrow points out the height which is same one marked by the arrow in **d**

Solar active region 11158, appeared on 2011 February, produced one X and several M class flares^23^ one of which is M6.6 class flare being involved in the solar eruption on 13 February, eventually producing CME as seen in Figs. 1 and 2. Figure 3a shows the photospheric magnetic field of the AR NOAA 11158 which produced the M6.6-class flare, taken by a Helioseismic and Magnetic Imager (HMI)^24^ onboard the Solar Dynamics Observatory (SDO)^25^. The 3D magnetic field lines approximated with the NLFFF are displayed in Fig. 3b, which are extrapolated from the photospheric magnetic field. Full view of the photospheric field and the NLFFF, and its comparison with an extreme ultraviolet (EUV) 171 Å image taken by the Atmospheric Imaging Assembly (AIA)^26^) on board SDO are shown in Supplementary Fig. 1. Note that the NLFFF is performed in a region surrounded by dashed square shown in Fig. 3a, while the magnetic field on boundary outside of the dashed-square is fixed to the potential field to avoid any small noise in the data introducing numerical errors^27,28^. Nevertheless, the reconstructed field is in good agreement with image taken in EUV (Supplementary Fig. 1) and satisfies better force-free condition (see Supplementary Fig. 2). The field lines are drawn with the value of the current density |**J**| superimposed and it can be seen that the twisted field lines residing in the central area of the AR have the strong current density.

Figure 3c focuses on the twisted field lines residing in the central area where the strong |**J**| is carried. These field lines sandwich the strong current density as shown in the small inset, which is favorable configuration to tether-cutting reconnection^29,30^. Note that the NLFFF is quite stable in the case where no external force is applied. This can been seen from the MHD simulation shown in Supplementary Fig. 3. Several studies of this event have shown it to be consistent with the tether-cutting reconnection model^31–34^. According to them, we induce the tether-cutting reconnection in the NLFFF, that can be achieved numerically through the MHD relaxation process^27,28^ (see Methods section), consequently, the reconnection can make long twisted lines as shown in Fig. 3d. The final state is then deviated from the force-free state^27,28^.

In order to address the physical state of the magnetic field before and after the tether-cutting reconnection, we first estimate the magnetic twist^35^ (*T*_w_: see Methods section) in each state. Figure 3e, f show scatter plots of all field lines on the parameter space *T*_w_ vs. |*B*_*z*_|, which is the magnetic field intensity on footpoint for the states before and after the tether-cutting reconnection, respectively. After the reconnection, few field lines are over one-turn but these are not likely to result in the kink instability because the twist values are far from the required threshold, *T*_w_ ≥ 1.5–2.0^36^.

The color of the field lines and vertical cross section in Fig. 3c, d corresponds to the decay index value. Even though the twisted lines shown in the NLFFF cannot reach the critical range of the instability as shown in Fig. 3c, the long twisted lines created through the tether-cutting reconnection have a possibility to be brought into the torus unstable area, i.e., loss of equilibrium area^37,38^. Therefore, the tether-cutting reconnection might be important to destroy the force-free state. However, from Fig. 3g exhibiting the height profile of the decay index measured along the dashed line in Fig. 3d, two unstable areas where *n* ≥ 1.5 exist. This is due to the quadrupole field, which results in a stable layer, found to be lie between them, i.e., saddle-like decay index profile is shown here. The each arrow in Fig. 3d, g points out the same height. Although the tether-cutting reconnection brings the twisted field lines into the lower area where they lose equilibrium, the eruption might be suppressed or terminate in the TI-stable layer^39^. In this case, however, the CME was clearly observed accompanying the M6.6 class flare as seen in Fig. 2; therefore, the question is raised of how to drive the flux tube eruption, leading to the CME, in the saddle-like decay index profile.

### Three-dimensional dynamics of the eruptive flux tube

**Fig. 4:**
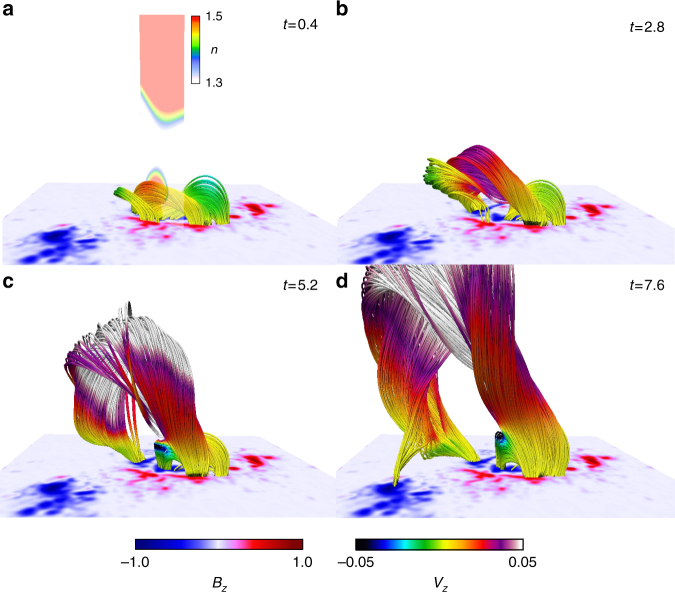
Temporal evolution of the three-dimensional dynamics of the eruptive flux tube. **a**–**d** Temporal evolution of the dynamics of the eruptive flux tube, together with *B*_*z*_ distribution at bottom. The color of the field lines corresponds to the normal velocity component. The vertical cross section in **a** is drawn with the decay index value in range from 1.3 to 1.5

In order to address the question, we perform MHD simulations starting with the magnetic fields just after the tether-cutting reconnection, as seen in Fig. 3d. Figure 4a–d exhibits the temporal evolution of the magnetic fields. The color of the field line indicates the vertical velocity component and the vertical cross section as seen in Fig. 4a corresponds to the decay index value in a range from 1.3 to 1.5. We first found that the small twisted flux tube surrounded by sheared field lines in early phase at *t* = 0.4, crosses the unstable (loss of equilibrium) area due to the tether-cutting reconnection. This consequently has small upward velocity while the TI-stable region exists above. Nevertheless, the flux tube ascends and further merges with the sheared field lines on both sides via the reconnection. Consequently, the strongly twisted flux tube is formed and then the top is accelerated over *V*_*z*_ = 0.05. From these results we found that during the eruption the large flux tube is automatically formed through the reconnection of the twisted field lines formed initially through the tether-cutting reconnection and the sheared field lines are mostly composed of the moderately twisted lines shown in the NLFFF. The highly twisted flux tube is then formed and continuously ascends without halting. Detailed dynamics is shown in Supplementary Movie 1.

**Fig. 5:**
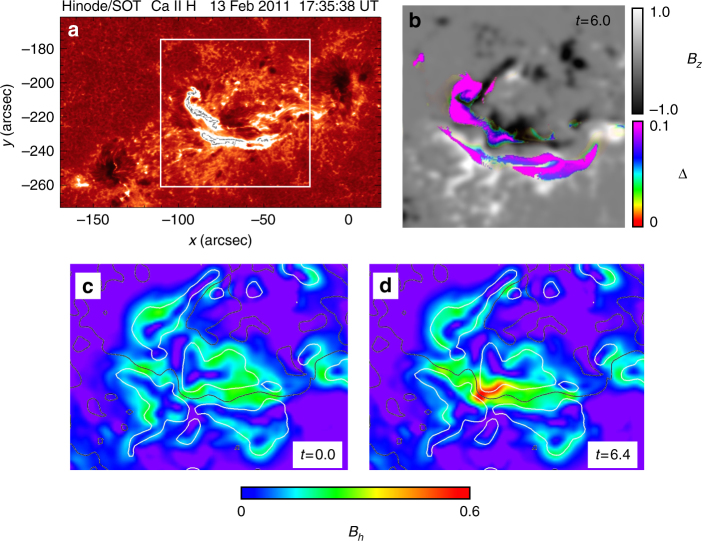
Comparison of simulation results with observations. **a** Flare ribbons during M6.6 solar flare, observed by Hinode at 17:35 UT on 2011 February 13. The intensity scale is 0–2000 DN. **b** Synthetic flare ribbons measured from total displacement of the field line footpoints^27^ superimposed on *B*_*z*_ distribution. The area corresponds to one surrounded by white square in **a**. **c**, **d** *B*_h_ distributions obtained from simulation, just prior and during the eruption, respectively. Black and white lines correspond to polarity inversion line and contour of |*B*_*z*_| = 0.25

We then compared the results of our simulation with observations to confirm the reliability of our simulation. Figure 5a shows flare ribbons observed by Hinode during the M6.6 flare, while synthetic flare ribbons^27,28^ produced from our simulation are shown in Fig. 5b. These synthetic ribbons correspond to total displacement of the footpoints of the field lines (Δ) during the eruption, i.e., the displacement is due to the reconnection. The synthetic ribbons are seen to be agreement with those seen in the observations. In Fig. 5c, d, we further show the horizontal field *B*_h_ defined as $\sqrt {B_x^2 + B_y^2}$ from which we found that it strongly enhances at the local area on the PIL. This result is consistent with previous observational studies^31^. Therefore, these results would support the reliability of our simulation. These temporal evolutions are shown in Supplementary Movies 2 and 3.

**Fig. 6:**
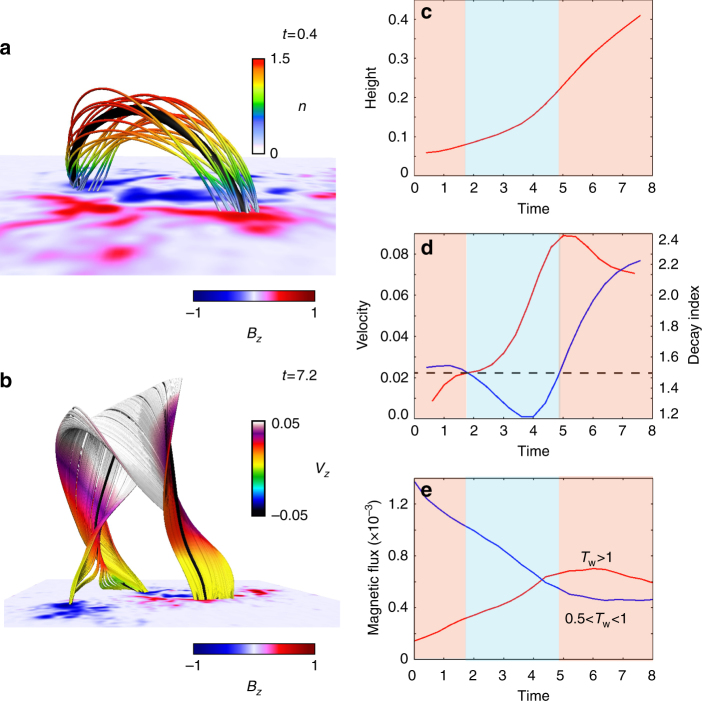
Quantitative analysis of the temporal evolution of the the eruptive flux tube. **a** The 3D field lines structure at *t* = 0.4 where the color indicates the decay index value in a range from 0 to 1.5. The particle locating at the point (0.4686, 0.51420, 6.00 × 10^−2^)L^*^ on a top of a black thick line is temporally traced. This field line is initially in non-equilibrium because this locates in an area satisfying *n* > 1.5, and eventually takes part in the large flux tube formed at late time. **b** 3D field lines at *t* = 7.2. The back thick line corresponds to the traced line. **c** Temporal evolution of the hight of the selected point of the traced line. **d** Temporal evolution of the velocity in red and the decay index in blue measured at each traced point. The dashed line marks the decay index value, *n* = 1.5. The red and blue masks mark regions, *n* ≥ 1.5 and *n* ≤ 1.5, respectively. **e** Temporal evolution of magnetic flux with more than one-turn in red and less than one-turn (but more than a half-turn) in blue

In order to interpret the dynamics quantitatively, we trace the temporal evolution of the physical values on a loop top of a selected twisted line plotted in thick black shown in Fig. 6a. The black line is in a region satisfying *n* ≥ 1.5 in the very earlier time *t* = 0.4, i.e., it has already lost the equilibrium and eventually takes part of the strongly twisted flux tube as seen in Fig. 6b. Figure 6c shows the temporal evolution of hight of the selected point from which we found increases as time passes. Figure 6d exhibits the temporal evolution of the velocity in red, and the decay index in blue which is measured at each time and each point. The decay index is over the critical value of the TI (*n* ≥ 1.5) during *t* ≈ 0.0–2.0 and after *t* ≈ 5.0, that is well capturing saddle-like profile. The initial state is already in a region satisfying *n* ≥ 1.5, therefore the upward velocity rapidly increases due to its non-equilibrium. When the selected loop flows into a region satisfying *n* ≤ 1.5, the velocity is then suppressed as expected. However, surprisingly, it is immediately accelerated even in the same area on contrary to expectation.

We then further look into the temporal evolution of the reconnected flux. Because the driving process might be related to the magnetic reconnection. That is a reason why the reconnection, which is caused by the ascending flux tube, of the moderately twisted lines can create the strongly twisted lines, i.e., it can supply the magnetic helicity into the ascending flux tube. Consequently, a more highly twisted flux tube, which would further enhance the upward hoop force^40^, is buildup. Therefore, it is important to identify how many of the strongly twisted field lines are created during the eruption. Figure 6e shows the temporal evolution of the magnetic flux with more than one-turn in red and less than one-turn in blue (but more than half-turn), respectively. We clearly found that the flux with highly twisted lines constantly increases during the time when the flux tube velocity is also increasing. On the other hand, the flux with moderately twisted lines decreases in quite opposite behavior to the highly twisted flux. This result implies that the highly twisted lines would be converted through the reconnection of the moderately twisted lines during the eruption. However, its still unclear whether the reconnection is really crucial for driving the eruption or not.

### Flux tube evolution and reconnection

**Fig. 7:**
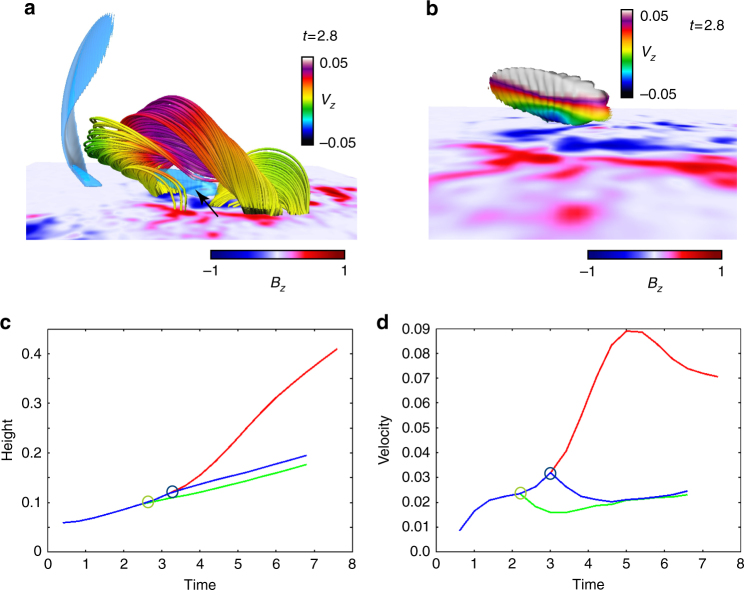
Hypothetical experiments for forcibly suppressing the reconnection. **a** Three-dimensional field lines at *t* = 2.8 which are identical format to those in Fig. 4 except the blue surface corresponds to the isosurface of |**J**|/|**B**| = 150. **b** A closeup of the surface, indicated by arrow in **a** drawn by *V*_*z*_ distribution. Reconnection during the eruption is suppressed here setting all velocity components to zero. **c** The temporal evolution of the height profiles obtained from each calculation. Green, and blue lines are obtained from the hypothetical experiments in which the reconnection is suppressed at *t* = 2.0 and 2.8, respectively, while the red line is obtained from the original calculation, i.e., the reconnection is not suppressed. **d** The temporal evolution of the velocity profiles obtained from each calculation. The format is identical to **c**

In order to clarify the role of the reconnection, we perform a hypothetical experiment in which during the eruption the reconnection is forcibly suppressed on the strong current density^41^ at *t* = 2.0 and 2.8, respectively. Figure 7a shows the 3D field lines at *t* = 2.8 starting acceleration toward the upper corona under which a strong current density is formed. The suppression of the reconnection is executed by forcibly setting all velocity components to zero on the |**J**|/|**B**| ≥ 150 formed under the flux tube highlighted by the black arrow shown in Fig. 7a. Note that all of the reconnection cannot be suppressed because reconnection can be allowed numerically on the current region with less than |**J**|/|**B**| = 150, in other words, if we halt the velocity in the area |**J**|/|**B**| ≤ 150, the spurious instability comes due to artificial inhabitation-process. Figure 7b focuses on the isosurface of |**J**|/|**B**| = 150, indicated by arrow in Fig. 7a. The color is specified by the *V*_*z*_ distribution from which we found the reconnection outflows. Since the reconnection in- and outflow are suppressed on this current sheet, the reconnection occurred here would be suppressed.

The temporal evolution of the height and velocity profiles are shown in Fig. 7c, d where the green and blue lines are obtained from the hypothetical experiments while the red line is derived from the original calculation, i.e., where reconnection is not suppressed. These results show that the height and velocity evolution, both of which are dramatically held down when the reconnection is suppressed while the flux tube shows slow ascecension even under the limited reconnection. We also reach another conclusion that the breakout reconnection^42^ does not dominate in this event because of poor acceleration even though the strong current region is formed above flux tube due to the quadrupole magnetic configuration where the velocity is not set to zero. Therefore the reconnection taking place under the flux tube largely contributes to the driving of the eruption. However, the physical process is not yet clear, *e*.*g*., the reason for that the reconnection can allow the flux tube to further accelerate in the TI-stable region.

**Fig. 8:**
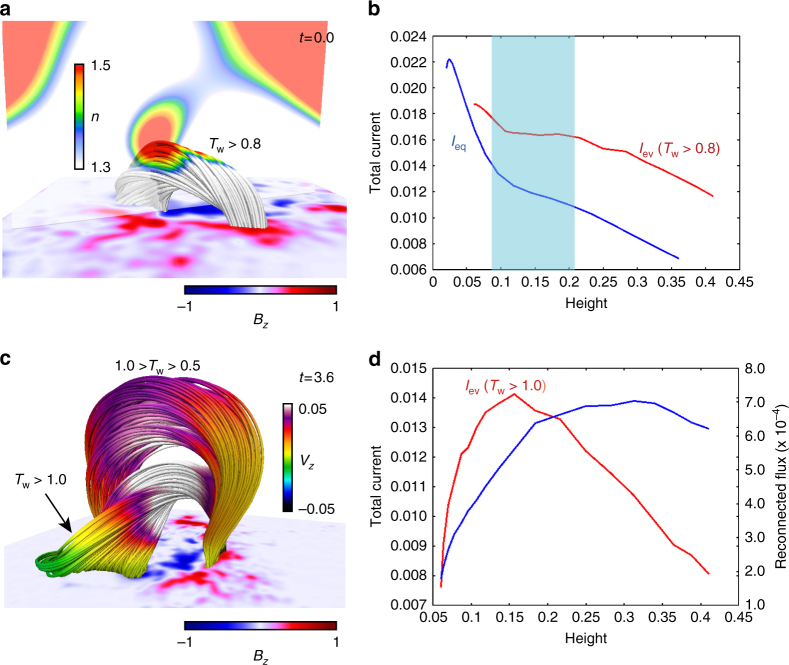
Evolution of the total Current in the Evolving Flux Tube. **a** The 3D field lines, all of which are twisted lines satisfying *T*_w_ ≥ 0.8, at *t* = 0.0 (just after the tether-cutting reconnection) and the vertical cross section drawn with the decay index *n* in the range from 1.3 to 1.5. The bottom shows the *B*_*z*_ distribution. **b** The red line corresponds to an evolution of the total current flowing in the evolving twisted flux tube, which is defined as a cluster of the twisted lines with *T*_w_ ≥ 0.8. The blue line shows the evolution of the current flux *I*_*eq*_ determining the equilibrium state of the flux tube at each hight. The blue mask corresponds to the region where *n* ≤ 1.5 satisfies during which the velocity increases as seen in Fig. 6. **c** The 3D field lines, those are highly (*T*_w_ ≥ 1.0) and moderately (1.0 > *T*_w_ ≥ 0.5) twisted lines, at *t* = 0.36 represented with *V*_*z*_. **d** The evolution of the total current with height, flowing in the evolving highly twisted flux tube (*T*_w_ ≥ 1.0) in red and the magnetic flux in blue, respectively

In order to reveal this, we clarify a relationship between the total current flowing in the flux tube and the reconnection. The total current is important to determine the equilibrium and dynamics of the flux tube^37,38^. Figure 8a shows the selected 3D field lines at *t* = 0.0 just after the tether-cutting reconnection all of which are dominated with the twisted lines with *T*_w_ ≥ 0.8. Since the NLFFF has no twisted lines over *T*_w_ ≥ 0.8, these are created by the reconnection and lose the equilibrium because they are in the torus-unstable area, *n* ≥ 1.5. We trace the thick black line described in Fig. 6a. Figure 8b plots the total current (*I*_ev_) as a function of the height, flowing in the flux tube which is a cluster of twisted lines with *T*_w_ ≥ 0.8. *I*_eq_ is defined as total current keeping the force balance of the hoop and strapping forces due to the flux tube current and the potential field^37^ (See method). If a flux tube rises up due to a loss of stability or equilibrium under the perfect conservation of magnetic helicity, then the total current decreases with the height because of the volume expansion^37,43^. Furthermore, *I*_ev_ curve might have a possibility to touch to *I*_eq_ one in TI-stable area and then another equilibrium might be achieved again^44^. Although the value of *I*_ev_ obtained form our simulation also shows a fast decrease in very early times, soon afterwards it, interestingly, keeps a saturation, shown in the blue highlighted region. This corresponds to the TI-stable area in which the velocity is dramatically accelerated as seen in Fig. 6d.

We show the 3D magnetic structure at *t* = 3.6, where *V*_*z*_ is highlited on the field lines in Fig. 8c. We found that the newly and strongly twisted lines with *T*_w_ ≥ 1.0 formed through reconnection drives the eruption, which have high upward velocity. We again show a plot for the magnetic flux with *T*_w_ ≥ 1.0 in blue line but as a function of the height in Fig. 8d. This flux corresponds to the reconnected flux from which we can suggest the reconnection creating the highly twisted flux tube (*T*_w_ ≥ 1.0) occurs efficiently until the flux tube can reach by h ≈ 0.2, then the total current flowing there, plotted in red, also dramatically increases. This result shows that the current enhancement would work efficiently even in TI-stable region, consequently, driving the eruption further and feeding back to the reconnection. After the flux tube passes at *h* ≈ 0.2, it simply expands without lots of reconnection, resulting in the current, *I*_ev_, decreasing.

## Discussion

Our results indicate that, once the system becomes unstable or loses the equilibrium, the dynamics are hardly influenced by a decay index profile, rather by a nonlinear process caused by the flux tube evolution and reconnection. We first quantitatively prove the eruption scenario for which the flux tube evolution enhances the reconnection which feeds back to make more largely and highly twisted flux tube, resulting into being further accelerated. We found that this cyclic process can show drastic eruption even in TI-stable area, i.e., this can well explain the observed eruption even in saddle-like decay index profile^18,19^.

**Fig. 9:**
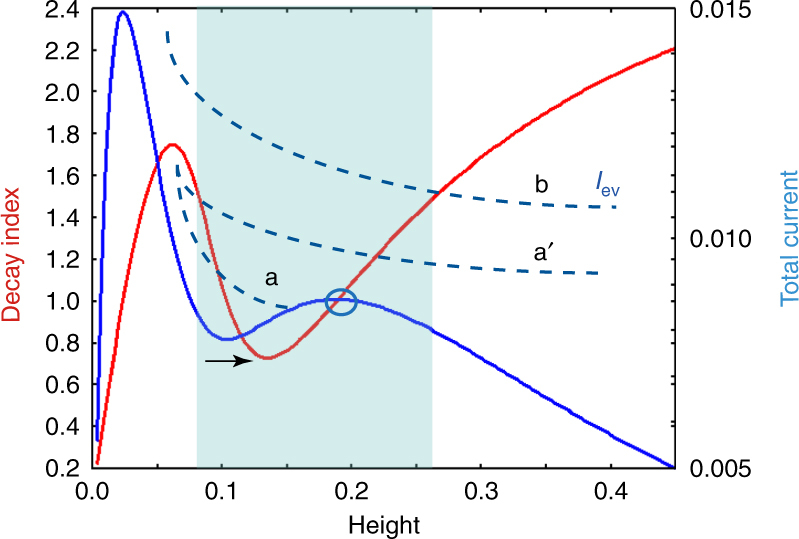
Decay index and total current flowing inside of the flux tube, plotted as a function of height. Red and blue lines show the decay index and total current of the flux tube taking the equilibrium as a function of height. These are measured along the dotted line shown in Fig. 3d, i.e., the decay index corresponds to Fig. 3g. Solid black arrow indicates the local minimum value of the decay index and blue circle points out the local maximum of the total current. A region highlighted by blue square corresponds to one satisfying *n* ≤ 1.5. Dashed blue lines (a, b and a′) are assumed as evolutions of the dynamic current *I*_ev_

However, observations^19^ show not only eruptive phenomena but also failed eruptions suggesting that the value of the decay index’s local minimum is a key issue. This might be explained by the behavior of the equilibrium line. Figure 6d shows decay index profile measured at the traced point, where the local minimum is around 1.2 which is consistent with observations showing eruptions^19^. Figure 9 shows decay index profile corresponding to one in Fig. 3g. This profile shows a deeper local minimum (around 0.75) pointed out by solid black arrow and the equilibrium line is prepared in same manner (see method) is plotted. We then found the local maximum in the equilibrium line in TI-stable area (*n* ≤ 1.5) as highlighted by blue circle which is not seen in Fig. 6d and quite opposite to the decay index profile. Therefore, in some cases, the dynamic current, *I*_ev_, might take a track following dotted blue line “a” and eventually meet the equilibrium line, i.e., a new equilibrium is then achieved^44^ while a flux tube can show an eruption if *I*_ev_ can track on the line “b”. On the other hand, as shown in this study, if reconnection creating the highly twisted lines is allowed during the eruption, *I*_ev_ might evolve along the line “a′” shifted from “a” and shows eruption. But, we require a more detailed theoretical study to investigate the conclusion further, and wish to leave as future works.

In summary, we found that the largely and highly twisted flux tube is buildup during the eruption and first proved eruptive scenario derived from nonlinear dynamics of flux tube evolution and reconnection. This can theoretically explain eruptions in saddle-like decay index profile as shown in several observations, in addition, we found the flux tube can be accelerated in TI-stable area. An interesting future test of this mechanism would be the X2.2 flare which occurred the following day^27,28^. We intend to investigate whether this same mechanism can explain this second eruption in the same active region in the near future.

## Methods

### Diagnosis of stability of the magnetic field

The decay index^10^ is defined as

1 $$n = - \frac{z}{{B_{{\mathrm{p}}_{\mathrm{t}}}}}\frac{{\partial B_{{\mathrm{p}}_{\mathrm{t}}}}}{{\partial z}},$$

where $B_{\mathrm{p}_{t}}$ denotes the horizontal component of the external field surrounding the flux tube. We here assume the potential field. This value is a proxy of the torus instability. If *n* satisfies the condition *n* ≥ 1.5, then the torus instability works on the flux tube, i.e., the upward hoop force is dominant over the strapping force from the external force.

The magnetic twist^35^ is defined as

2 $$T_{\mathrm{w}} = \frac{1}{{4\pi }}{\kern 1pt} {\int} {\kern 1pt} \frac{{\nabla \times {\mathbf{B}} \cdot {\mathbf{B}}}}{{\left| {\mathbf{B}} \right|^2}}{\mathrm{d}}l,$$

where d*l* is a line element of a field line. Note that in this study the twist is calculated satisfying the condition 25*G* > *B*_*z*_ within the dashed square shown in Fig. 3a, in order to exclude the contributions from the weak horizontal magnetic fields on the photosphere. This formulation means turns of two infinitesimally close filed lines each other. However, this is also good proxy of kink instability and applicable to several active region^22,45^. In this study we define a flux tube using this twist value because we need to detect the twisted field lines *T*_w_ ≥ 0.8 driving the eruption at each time. If we wish to trace the detailed variations of field lines and hyperbolic flux tube structure^46^, squashing degree method would be useful^46^. However, since these detailed variations are not discussed here, we simply defined the flux tube using magnetic twist value.

### Numerical method

Both of our NLFFF extrapolation method and MHD simulation are based on the zero-beta MHD approximation^27,47^ as following,

3 $$\rho = \left| {\mathbf{B}} \right|,$$

or

4 $$\frac{{\partial \rho }}{{\partial t}} = - \nabla \cdot (\rho {\mathbf{v}}) + \nu _\rho \nabla ^2(\rho - \rho _0),$$

5 $$\frac{{\partial {\mathbf{v}}}}{{\partial t}} = - ({\mathbf{v}} \cdot \nabla ){\mathbf{v}} + \frac{1}{\rho }{\mathbf{J}} \times {\mathbf{B}} + \nu \nabla ^2{\mathbf{v}},$$

6 $$\frac{{\partial {\mathbf{B}}}}{{\partial t}} = \nabla \times ({\mathbf{v}} \times {\mathbf{B}} - \eta _{\mathrm{i}}{\mathbf{J}}) - \nabla \phi ,$$

7 $${\mathbf{J}} = \nabla \times {\mathbf{B}},$$

8 $$\frac{{\partial \phi }}{{\partial t}} + c_{\mathrm{h}}^2\nabla \cdot {\mathbf{B}} = - \frac{{c_{\mathrm{h}}^2}}{{c_{\mathrm{p}}^2}}\phi ,$$

where the subscript *i* of *η* corresponds to ‘NLFFF’ or ‘MHD’. The length, magnetic field, density, velocity, time, and electric current density are normalized by L* = 216 Mm, B* = 2500 G, ρ* = |B*|, $V_{\mathrm{A}}^ \ast \equiv B^ \ast {\mathrm{/}}(\mu _0\rho ^ \ast )^{1{\mathrm{/}}2}$, where *μ*_0_ is the magnetic permeability, $\tau _{\mathrm{A}}^ \ast \equiv L^ \ast {\mathrm{/}}V_{\mathrm{A}}^ \ast$, and J* = B*/*μ*_0_L*, respectively. *ρ*_0_ is initial state of the plasma density and *ϕ* is the convenient potential for cleaning errors derived from $\nabla \cdot {\mathbf{B}}$^48^, respectively. *ν* and *ν*_*ρ*_ are viscosity fixed by 1.0 × 10^−3^ and 1.0 × 10^−4^, and the coefficients $c_{\mathrm{h}}^2$, $c_{\mathrm{p}}^2$ in Eq. 8 also fix the constant values, 0.04 and 0.1, respectively. The initial condition of the density is given by *ρ* = |**B**| and the velocity is set at zero in all space in the NLFFF calculation and also MHD simulation.

A numerical box is designed by 216 × 216 × 216 (Mm^3^), which is given as 1 × 1 × 1 in its non-dimensional value, divided by 300 × 300 × 300 grid points. Photospheric magnetic field of AR11158 is shown in Supplementary Fig. 1a taken by SDO/HMI, observed at 16:00 UT on 13 February 2011, corresponding to 90 min approximately before the M6.6-class flare occurred at 17:28 UT. The photospheric magnetic field covers a 216 × 216 (Mm^2^) region, divided by a 600 × 600 grid which is provided by the description of CEA projected and remapped vector magnetic field^1^. It is obtained using the very fast inversion of the Stokes vector algorithm^49^ based on the Milne–Eddington approximation. A minimum energy method was used to resolve 180° ambiguity in the azimuth angle of the magnetic field^50,51^. A 2 × 2 binning of the original data yields the grid resolution in this study. The photospheric magnetic field is preprocessed according to Wiegelmann et al.^52^. Note that this preprocessing performs smoothing on the currents and decreases the free-energy from its original value, to satisfy more force-free state^53^.

### NLFFF extrapolation

The NLFFF extrapolation is executed in the local area (0.38 L* < *x* < 0.65 L*, 0.38 L* < *y* < 0.65 L*) surrounded by dashed square in Fig. 3a in which the observed transverse field is applied. The outside is fixed by the potential field in order to avoid an effect of less-accurate transverse field and keep more stable state^27,28^. Note that, in general, force-free extrapolation in a limited field-of-view decreases the accuracy of the state^54^. However, in this case the less-accurate transverse field existing outside of the active region might further negatively impact the final state. The NLFFF extrapolation employs pseudo density (Eq. 3) which is assumed to be proportional to |**B**| in order to make the Alfven speed relax in the space, and Eqs 5–8. The resistivity is given as follows,

9 $$\eta _{{\mathrm{nlfff}}} = \eta _0 + \eta _1\frac{{\left| {{\mathbf{J}} \times {\mathbf{B}}} \right|\left| {\mathbf{v}} \right|}}{{\left| {\mathbf{B}} \right|}},$$

where *η*_0_ = 5.0 × 10^−5^ and *η*_1_ = 1.0 × 10^−3^ in the non-dimensional unit. The second term is introduced to accelerate the relaxation to the force-free field particularly in weak field region. The potential field is given as the initial condition, which is extrapolated only from the normal component of the photospheric magnetic field^55^. During the iteration, three components of the magnetic field are fixed at each boundary while the velocity is fixed to zero and von Neumann condition ∂/∂*n* = 0 is imposed on *ϕ*. Note that the bottom boundary is fixed according to

$${\mathbf{B}}_{{\mathrm{bc}}} = \zeta {\mathbf{B}}_{{\mathrm{obs}}} + (1 - \zeta ){\kern 1pt} {\mathbf{B}}_{{\mathrm{pot}}},$$

where **B**_bc_ is the transverse component which is determined by a linear combination of the observed magnetic field (**B**_obs_) and the potential magnetic field (**B**_pot_). *ζ* is a coefficient in range from 0 to 1. When $R = {\int} {\kern 1pt} \left| {{\mathbf{J}} \times {\mathbf{B}}} \right|^2{\kern 1pt} {{\mathrm{d}V}}$, which is calculated over the interior of the computational domain, falls below a critical value denoted by *R*_min_ during the iteration, the value of the parameter *ζ* is increased to *ζ* = *ζ* + d*ζ*. In this paper, *R*_min_ and d*ζ* have the values 5.0 × 10^−3^ and 0.02, respectively. If *ζ* becomes equal to 1, **B**_bc_ is completely consistent with the observed data. Furthermore, the velocity is controlled as follows. If the value of v* becomes larger than *v*_max_ (here set to 0.01), then the velocity is modified as follows: ${\mathbf{v}} \Rightarrow (v_{{\mathrm{max}}}{\mathrm{/}}v^ \ast ){\mathbf{v}}$. These processes would help to avoid a sudden jump from the boundary into the domain during the iteration.

### MHD relaxation

In order to induce the tether-cutting reconnection, we further perform the MHD relaxation using the NLFFF as initial condition^27,28^, which is basically same as the NLFFF extrapolation method except replacing with the anomalous resistivity and the velocity limit are not imposed as shown in the NLFFF extrapolation. The anomalous resistivity is designed as

10 $$\eta = \left\{ {\begin{array}{*{20}{l}} {\eta _0} \hfill & {J < j_{\mathrm{c}},} \hfill \\ {\eta _0 + \eta _2\left( {{\textstyle{{J - j_{\mathrm{c}}} \over {j_{\mathrm{c}}}}}} \right)^2} \hfill & {J  >j_{\mathrm{c}},} \hfill \end{array}} \right.$$

where *η*_2_ = 5.0 × 10^−4^, and *j*_c_ is the threshold current, set to 35 in this study. Note that the values of these parameters take account for suitable calculation time and numerical stability, which depend on normalized resistivity and grid size. This process plays a role on creating higher twisted lines as shown in Fig. 3d (also see Inoue et al.^28^) than those in the NLFFF because the anomalous resistivity can enhance the reconnection locally between the twisted lines^56^. In this study, 1000 iterations are executed. Note that newly formed magnetic field via a tether-cutting reconnection is already deviated from the equilibrium state.

### MHD simulation

The MHD simulation composed of equations from (4) to (8) is carried out where the initial condition employs the magnetic field after the tether-cutting reconnection shown in Fig. 3d and the anomalous resistivity is replaced by the uniform resistivity *η*_mhd_ = 1.0 × 10^−5^. The density is initially given as *ρ* = |**B**| and the value is fixed at boundaries during the calculation. The normal component, **B**_h_, is fixed but the transverse component, **B**_t_, is variable according to the dynamics. Boundary conditions for other variables are identical to those in the NLFFF calculation.

### Equilibrium of the flux tube

In order to obtain an equilibrium line, i.e., total current *I*_eq_ of the flux tube achieving equilibrium state, the simple environment is assumed that the current is uniformly distributed in circular-shape flux tube and a low aspect ratio is assumed which is less than one. In general, the sigmoidal-shape is the typical magnetic structure prior to flaring, which is far from a circular-shape flux tube model demonstrated here. However, we focus on the magnetic field after the tether-cutting reconnection and already in slow rising phase. The writhe and sigmoidal structure then would be expected to be reformed to circular-shape flux tube^29,30^ and it can be seen in Fig. 8a. Therefore, we use the theoretical model for this analysis. The force balance can be described as following^37^

11 $$I_{{\mathrm{eq}}}^2r_{\mathrm{c}} - \left| {{\mathbf{B}}_{{\mathrm{p}},{\mathrm{t}}}} \right|I_{{\mathrm{eq}}} = 0,$$

where

12 $$r_{\mathrm{c}} = \frac{1}{{4\pi h}}\left( {{\mathrm{ln}}\left( {{\textstyle{{8h} \over a}}} \right) - 1 + {\textstyle{{l_{\mathrm{i}}} \over 2}}} \right),$$

**B**_p,t_ corresponds to the horizontal component of the potential field, and *h*, *a* and *l*_i_ denote a height of the torus, the minor radius, and the internal inductance, respectively. In this study we trace the black solid line shown in Fig. 6a, then |**B**_p,t_| can be calculated at each heigh, *h*. Under the assumption *l*_i_ takes 0.5 and *a*/*h* = 0.5, which was estimated from Fig. 8a, was assumed^57,58^.

### Visualization

All of the 3D visualizations were done using VAPOR^57,58^.

### Data availability

The data that support the findings of this study are available from the corresponding author upon request.

## Electronic supplementary material

Supplementary Information

Description of Additional Supplementary Files

Supplementary Movie 1

Supplementary Movie 2

Supplementary Movie 3

## Footnotes

1
http://jsoc.stanford.edu/jsocwiki/ReleaseNotes
