# Supplementary material for: Formation and dynamics of a solar eruptive flux tube
Source: Nat Commun. 2018 Jan 12;9:174. doi: 10.1038/s41467-017-02616-8 (PMC5766525; doi:10.1038/s41467-017-02616-8)
Supplement: Supplementary file 1 — Supplementary Information [file 41467_2017_2616_MOESM1_ESM.pdf]

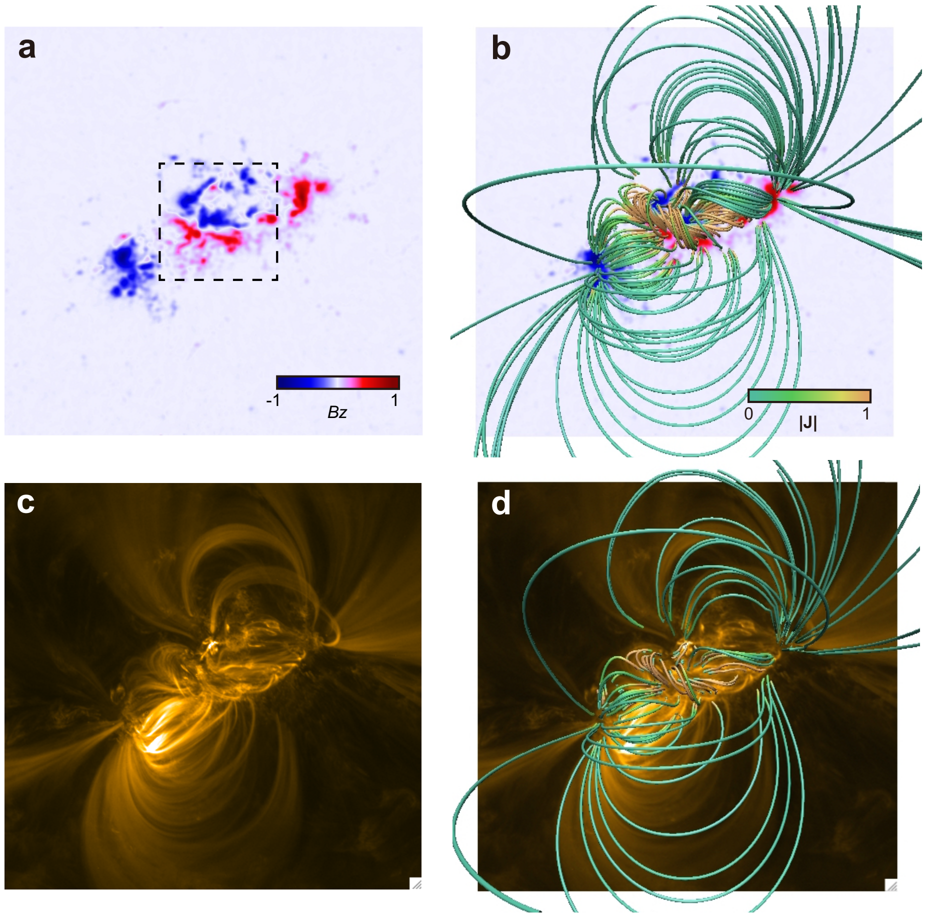


**Supplementary Figure** 1: **Full field of view of the photospheric field used in this study and the production of the NLFFF. a**, The full view of the photospheric magnetic field ($B_z$ distribution) used in this study, which was observed at 16:00UT corresponding to approximately 90 minutes before the M6.6 class flare. The NLFFF is executed only within the dashed square and the boundary of the outside is fixed by the potential field to exclude an inconsistent force-free $\alpha$ derived from the weak horizontal field. **b**, The magnetic field lines are shown in the NLFFF approximation. The color of the field lines corresponds to the value of the current density $|\mathbf{J}|$. **c**, EUV 171 Å image observed at 16:00 UT taken by AIA onboard the SDO. This image shows field lines frozen into the high temperature plasma, projected in 2D space. **d**, The NLFFF is superimposed on the EUV image.

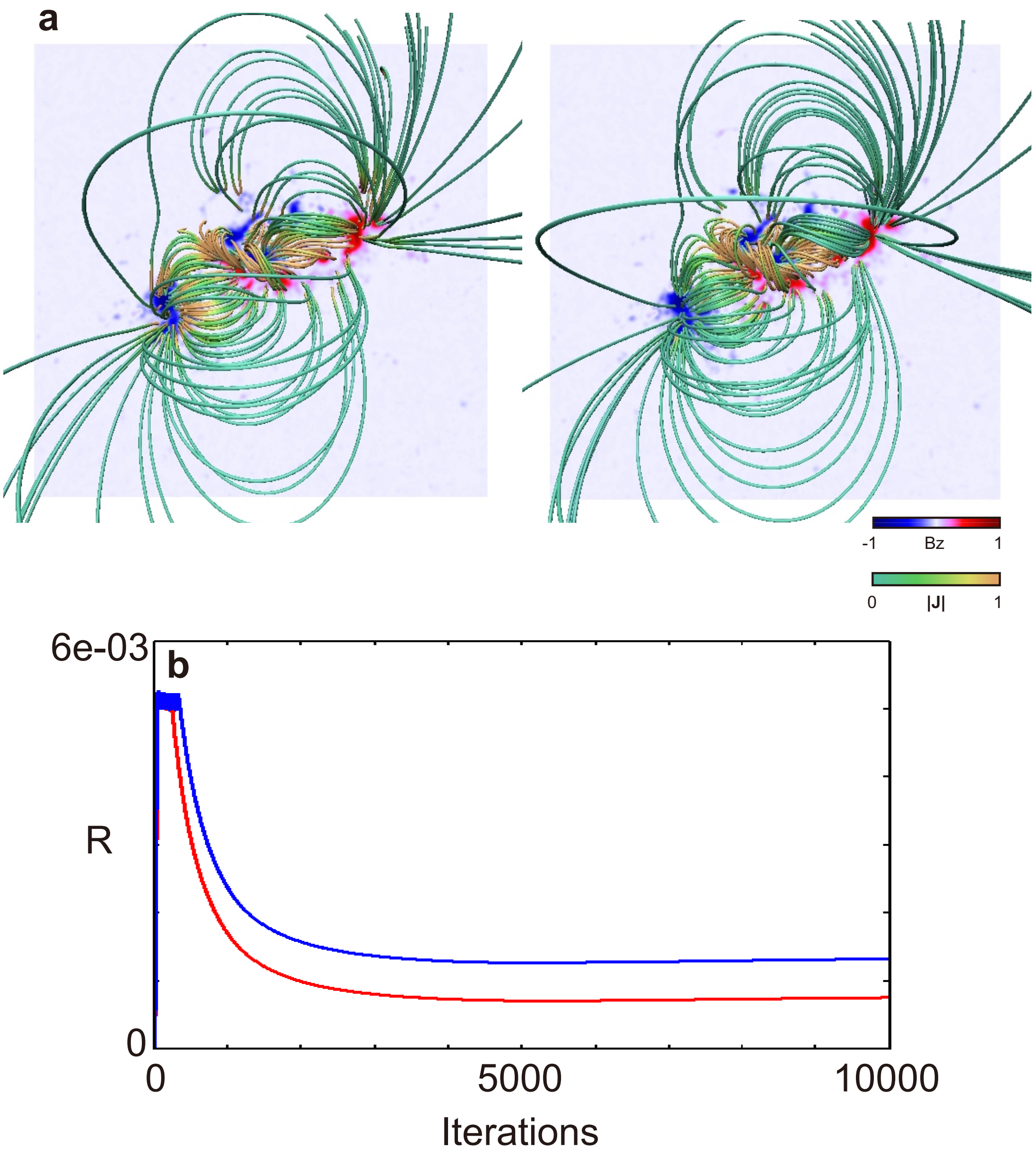


**Supplementary Figure** 2: **Comparing the NLFFF extrapolation from the photospheric field fully/partially applied. a**, The NLFFFs are shown in left and right panels, respectively, where the left one is extrapolated from the photospheric magnetic field fully applied whilst the right one is partially applied, *i.e.*, the NLFFF extrapolation is executed in the limited area focusing on the central area within the dashed square as seen in Fig. 3a or supplementary Fig. 1a. The format is identical to Fig. 3b. **b**, The iteration profile of $R = \int |\mathbf{J} \times \mathbf{B}| \mathrm{dV}$ for the NLFFF extrapolation fully applied to the photospheric magnetic field in blue and partially applied in red, where V is the whole volume of the numerical box except the each boundary.

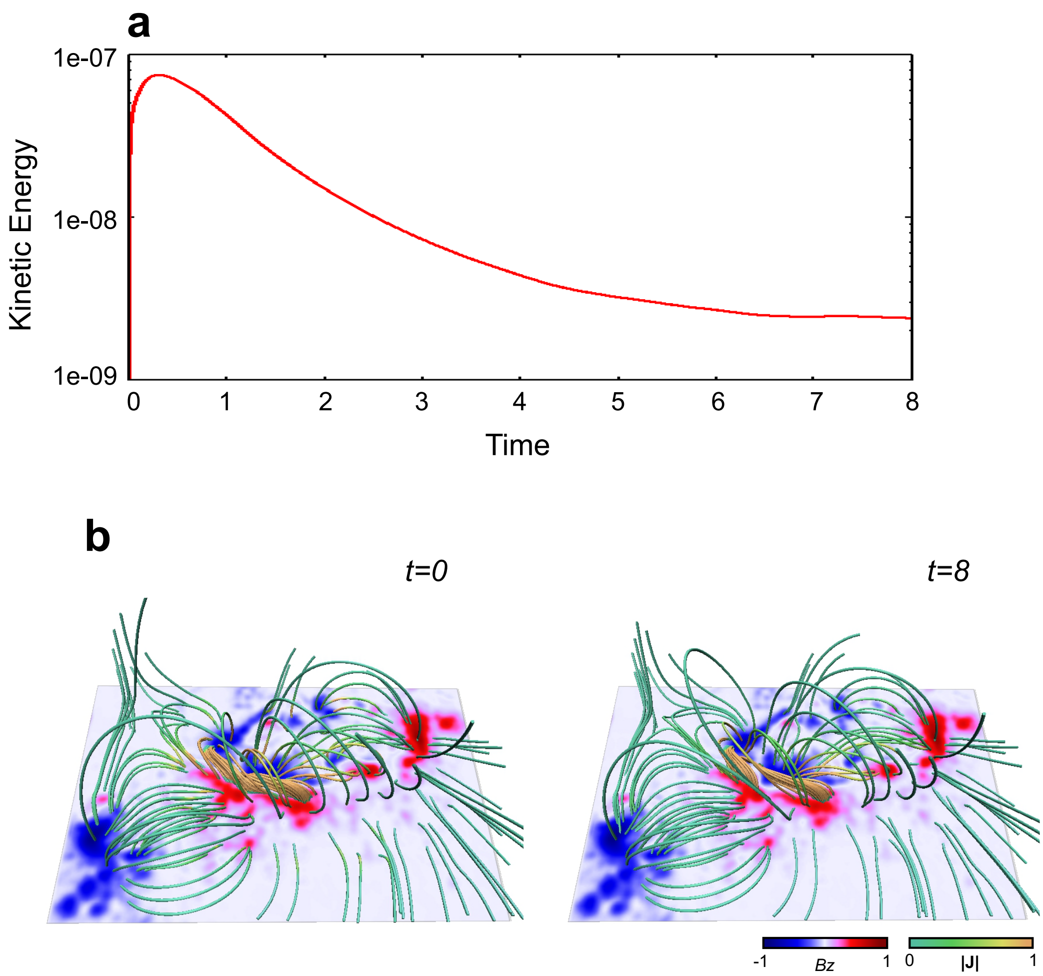


**Supplementary Figure** 3: **The MHD evolution starting with the NLFFF under no external force**. **a**, The temporal evolution of the kinetic energy in the MHD simulation initiating with the NLFFF under no external force. **b**, The snap shots of the 3D magnetic field lines highlighted with the current density $|\mathbf{J}|$ are plotted on $B_z$ distribution at initial ($t$=0) and last time ($t$=8), respectively. Although slight differences in these field lines are found, the eruption is not observed.
