# Supplementary material for: Formation and dynamics of a solar eruptive flux tube
Source: Nat Commun. 2018 Jan 12;9:174. doi: 10.1038/s41467-017-02616-8 (PMC5766525; doi:10.1038/s41467-017-02616-8)
Supplement: Supplementary file 2 — Description of Additional Supplementary Files [file 41467_2017_2616_MOESM2_ESM.pdf]

Description of Additional Supplementary Files

File Name: Supplementary Movie 1
Description: Temporal evolution of the magnetic field lines focusing on the eruptive twisted flux tube. The field lines are highlighted by Vz. Bz distribution is plotted on the bottom surface.

File Name: Supplementary Movie 2
Description: Temporal evolution of synthetic flare ribbon. This distribution corresponds to the total displacement of the reconnected field lines(Δ) during the eruption. The detailed manner is described in Inoue et al. ApJ 788, 182 (2014) and Inoue et al. ApJ 803, 73 (2015).

File Name: Supplementary Movie 3
Description: Temporal evolution of the horizontal magnetic field (Bh) measured on the photosphere during the eruption. Solid black and white lines correspond to polarity inversion line and contours of $|Bz| = 0.25$.
